# Developing an Artificial Intelligence Model for Reading Chest X-rays: Protocol for a Prospective Validation Study

**DOI:** 10.2196/39536

**Published:** 2022-11-16

**Authors:** Queralt Miró Catalina, Aïna Fuster-Casanovas, Jordi Solé-Casals, Josep Vidal-Alaball

**Affiliations:** 1 Unitat de Suport a la Recerca de la Catalunya Central Fundació Institut Universitari per a la Recerca a l'Atenció Primària de Salut Jordi Gol i Gurina Sant Fruitós de Bages Spain; 2 Health Promotion in Rural Areas Research Group Gerencia Territorial de la Catalunya Central Institut Català de la Salut Sant Fruitós de Bages Spain; 3 Data and Signal Processing group, Faculty of Science, Technology and Engineering University of Vic-Central University of Catalonia Vic Spain; 4 Department of Psychiatry University of Cambridge Cambridge United Kingdom; 5 Faculty of Medicine University of Vic-Central University of Catalonia Vic Spain

**Keywords:** artificial intelligence, machine learning, chest x-ray, radiology, validation

## Abstract

**Background:**

Chest x-rays are the most commonly used type of x-rays today, accounting for up to 26% of all radiographic tests performed. However, chest radiography is a complex imaging modality to interpret. Several studies have reported discrepancies in chest x-ray interpretations among emergency physicians and radiologists. It is of vital importance to be able to offer a fast and reliable diagnosis for this kind of x-ray, using artificial intelligence (AI) to support the clinician. Oxipit has developed an AI algorithm for reading chest x-rays, available through a web platform called ChestEye. This platform is an automatic computer-aided diagnosis system where a reading of the inserted chest x-ray is performed, and an automatic report is returned with a capacity to detect 75 pathologies, covering 90% of diagnoses.

**Objective:**

The overall objective of the study is to perform validation with prospective data of the ChestEye algorithm as a diagnostic aid. We wish to validate the algorithm for a single pathology and multiple pathologies by evaluating the accuracy, sensitivity, and specificity of the algorithm.

**Methods:**

A prospective validation study will be carried out to compare the diagnosis of the reference radiologists for the users attending the primary care center in the Osona region (Spain), with the diagnosis of the ChestEye AI algorithm. Anonymized chest x-ray images will be acquired and fed into the AI algorithm interface, which will return an automatic report. A radiologist will evaluate the same chest x-ray, and both assessments will be compared to calculate the precision, sensitivity, specificity, and accuracy of the AI algorithm. Results will be represented globally and individually for each pathology using a confusion matrix and the One-vs-All methodology.

**Results:**

Patient recruitment was conducted from February 7, 2022, and it is expected that data can be obtained in 5 to 6 months. In June 2022, more than 450 x-rays have been collected, so it is expected that 600 samples will be gathered in July 2022. We hope to obtain sufficient evidence to demonstrate that the use of AI in the reading of chest x-rays can be a good tool for diagnostic support. However, there is a decreasing number of radiology professionals and, therefore, it is necessary to develop and validate tools to support professionals who have to interpret these tests.

**Conclusions:**

If the results of the validation of the model are satisfactory, it could be implemented as a support tool and allow an increase in the accuracy and speed of diagnosis, patient safety, and agility in the primary care system, while reducing the cost of unnecessary tests.

**International Registered Report Identifier (IRRID):**

PRR1-10.2196/39536

## Introduction

Chest x-rays are currently the most commonly used type of x-rays, accounting for up to 26% of all radiographic tests performed [1-3]. This technique makes it possible to identify cardiopulmonary conditions, verify the correct positioning of devices such as pacemakers, gastric and thoracic tubes, or detect obstructed blood vessels, among others [4,5].

However, chest radiography is a complex imaging modality to interpret [6]. In fact, several studies have reported discrepancies in chest x-ray interpretations among emergency physicians and radiologists [7,8]. Therefore, it is of vital importance to be able to offer a fast and reliable diagnosis for this kind of x-ray, using artificial intelligence (AI) to support the clinician.

Radiology is one of the areas in which AI has had the greatest impact. Radiologists are medical professionals who use imaging technology to diagnose pathologies. Major advances in AI have enabled these professionals to make use of this tool to improve workflows and accuracy, thus reducing economic costs by avoiding unnecessary tests [5,9].

AI is a branch of computer science that aims to simulate tasks related to human intelligence, including processes such as learning and improvement through feedback or reasoning, using machines [10]. It is a tool capable of learning and analyzing large amounts of information, in different formats and at high speed, to aid in the accuracy and speed of diagnosis, facilitate and streamline clinical care, and support public health interventions, among many other applications [11,12]. The rapid growth of computer science and big data indicates that it is here to stay and will significantly change the practice of medicine [13].

The development of a computer system capable of interpreting thoracic x-rays as efficiently as a radiologist could be of great benefit in the clinical setting. The results of Rajpurkar et al’s [14] study on the application of deep learning for chest x-ray diagnosis presents an algorithm (CheXNeXt), which performs comparably with professionals in detecting multiple thoracic pathologies.

Wu et al [2] compares the interpretations of 5 radiology residents with those of an AI algorithm and corroborates that these well-trained techniques can achieve performance levels similar to professionals. Furthermore, Ciceró et al [15] demonstrates that convolutional neural networks can be trained with data sets to classify chest x-rays and obtain clinically useful performance in the detection and exclusion of common pathologies. 

Oxipit is one of the leading companies in medical image reading using AI, whose goal is to introduce advances in deep learning techniques into daily clinical practice [16]. The company has developed an AI algorithm for reading chest x-rays, available through a web platform called ChestEye.

This platform is an automatic computer-aided diagnosis system where the inserted chest x-ray is read and an automatic report is returned with a capacity to detect 75 pathologies, covering 90% of diagnoses. Thus, ChestEye allows radiologists to analyze only the most relevant x-rays [17,18].

Therefore, the main objective of the study is to perform a prospective validation of the ChestEye AI algorithm as a diagnostic decision support tool for the diagnosis of chest x-rays and to try to improve or optimize it if possible.

## Methods

### Design

A prospective study will be conducted to validate the AI algorithm, comparing the ChestEye AI diagnoses with the radiologists’ diagnoses, which is considered the gold standard. The process will include the following steps:

1. The patient will arrive at the primary care center for the chest x-ray, and if he/she meets the inclusion and exclusion criteria, the health care staff will briefly explain the study and provide the informed consent form to be signed.
2. Regardless of whether the user has agreed to participate in the study or not, the reference radiologist will perform the diagnosis of the x-ray to be entered into the Primary Care Clinical Station (ECAP). This station is the computerized clinical history program used by all professionals in the primary care network of the Institut Català de la Salut (ICS).
3. If the user has agreed to participate in the study, the researchers will extract the ECAP x-ray and enter it into the AI algorithm through their web-based platform to obtain their diagnosis.
4. Finally, the performance and fit of the AI model against the gold standard (radiologists’ diagnoses) will be validated and evaluated.

The AI algorithm ChestEye, from Oxipit, is an automatic and autonomous algorithm, without the involvement of the radiologist, which works through a web-based platform where the image is entered in DICOM format, and returns an image evaluation and diagnosis. The algorithm has the capacity to detect 75 pathologies, covering 90% of the diagnoses [16].

ChestEye has been previously developed and trained by Oxipit through iterative processing of large amounts of data by neural network-based AI algorithms, allowing the software to learn automatically from patterns or features in the data.

### Scope, Period, and Participants

The study will be performed at the ICS Primary Care Centre Vic Nord (Osona, Catalonia, Spain), a reference center where all chest x-rays in the region are performed. It is expected that data can be obtained in 5 to 6 months, from February 7, 2022, with recruitment using consecutive sampling. In June 2022, more than 450 chest x-rays have been collected, so it is expected that 600 samples will be gathered in July 2022.

The reference population of the prospective study will be the entire population of Osona due to undergo a chest x-ray at this center, with prior informed consent.

The study will include only anteroposterior chest x-rays performed from the beginning of the study until the necessary sample is obtained from patients with authorized informed consent and who are older than 18 years. Pregnant women and chest x-rays of inadequate quality (poor exposure, images not centered or rotated) will be excluded from the study as the AI algorithm needs high-quality images to maximize its performance.

### Sample Size and Sampling Procedure

To validate the AI algorithm, a total sample of 600 x-rays will be needed, 200 of them with one of the 75 pathologies detected by the AI algorithm. The proposed sample is based on calculations used in similar research [1,14,19,20]. Furthermore, it has been calculated that with this sample size, we can estimate global accuracy considered to be around 70% with 95% confidence, 4% precision, and an anticipated replacement rate of 15%.

### Data Collection and Information Sources

The ICS health care personnel performing the chest x-rays will explain the study and its objectives to the users, and will give the patient an information sheet, together with the informed consent form, to all those who meet the inclusion criteria. The ICS Central Catalonia technical service will then extract all these x-rays with their corresponding diagnosis. Each x-ray will be associated with a unique identifier to relate it to its diagnosis and eliminate any nonanonymized information. Next, the study’s principal researchers will input the x-rays into the AI system to obtain the diagnoses of the models using the algorithm. Finally, the data will be analyzed by comparing the diagnoses of the practitioner and the algorithm.

### Data Analysis

To validate the algorithm, the results using the AI algorithm and the diagnoses made by radiologists will be compared. With this, the confusion matrix of the algorithm will be obtained from the correctly classified positive (TP), correctly classified negative (TN), false positive (FP), and false negative (FN) x-rays. The sensitivity, specificity, classification rate (accuracy), and area under the curve (AUC) of the algorithm will be calculated from this matrix. These results can be obtained for each pathology and the classifier as a whole. Accuracy, recall, and F-measurement will also be calculated for the overall classifier and each pathology.

To evaluate the classifier for multipathology radiology, the data will be treated as a set of binary variables, one for each pathology. In this case, the AUC will be calculated using the One-vs-All method. Macroaveraging and microaveraging measures will be considered to highlight pathologies with lower prevalence. The data will be analyzed with the statistical software R (version 4.1.2; R Foundation for Statistical Computing), whose intervals will be of 95% confidence, with a significance level of 5%.

### Ethics Approval

The University Institute for Research in Primary Health Care Jordi Gol i Gurina (Barcelona, Spain) ethics committee approved the trial study protocol (approval code: 21/288). Written informed consent will be requested from all patients participating in the study.

## Results

Patient recruitment began in February 2022, and it is expected that data can be obtained in 5 to 6 months. On June 2022, more than 450 chest x-rays have been collected, so it is expected that 600 samples will be gathered in July 2022. Each user who agrees to participate in the study will be asked for written informed consent and will be given the project information sheet. Data collection for all participants is expected to be completed by June 2022, and the results can be published by the end of 2022.

In this way, we hope to obtain sufficient evidence to demonstrate that the use of AI in the reading of chest x-rays can be a good tool for diagnostic support. However, in the context of Central Catalonia (the Catalan region where the data was collected), there is an increasingly lower volume of radiologists, and therefore, tools need to be developed to support professionals who have to interpret these tests [21,22].

Once the algorithm has been validated, the values of sensitivity, specificity, accuracy, and AUC will be used to evaluate the results obtained and to determine whether it would be a good model to be introduced in the Catalan health system.

## Discussion

### Comparison With Prior Work

The protocol of this study aims to perform a prospective validation of an AI algorithm and to demonstrate that the use of AI in chest x-rays can become a good tool for supporting professionals in their diagnoses. In this context, this study may bring added value for both patients and primary care physicians as it will provide information about the effectiveness of the AI algorithm and its limitations. External validation of new AI tools is essential before implementing them as diagnostic systems.

Studies are showing that the application of AI models can be comparable to the performance of a professional in the detection of multiple pathologies [2,14,15]. However, before committing resources to AI applications in health care, the acceptance of these applications should be studied. Although some studies have shown that AI has a high potential to be useful as a diagnostic tool, it is remarkable that most patients still preferred the diagnoses done by physicians, and professionals only accepted AI models if they were used in combination with “human diagnosis” [23,24]. In this context, leading health care systems are moving toward the digitization of health care. Therefore, it is time to provide and validate tools that can enable improvement in the workflow of professionals as well as support their diagnosis. Always consider the clinical context for the subsequent application of these tools.

Furthermore, it has to be taken into consideration that most of the AI studies conducted in health care were just proof-of-concept projects that used retrospective clinical data sets [25]. The application of AI techniques in the real clinical context is becoming more and more relevant to ensure its safe adoption in health care systems. Thus, this study will be conducted using prospective data sets, promoting the health care AI researchers’ community to work closely with health care providers in a real clinical environment.

### Limitations

This study has some limitations. The most relevant one is that there is the possibility of not obtaining a homogeneous distribution across the 75 possible diagnoses due to their low prevalence. In that sense, as a large number of diseases can be detected by chest x-ray, we will probably not obtain representative results for the less prevalent diseases. As class imbalance may be a limitation, the F score will be evaluated. Otherwise, the large number of more frequent pathologies may overestimate the quality of the algorithm (accuracy, sensitivity, and specificity). Another possible limitation is that a small amount of sample is likely to be lost due to inadequate image quality, as chest x-rays of inadequate quality will be excluded.

### Conclusions

If the results of the model validation are satisfactory, the model can be implemented as a support tool and can increase diagnostic accuracy and speed, patient safety and agility within the primary care system, and reduce unnecessary testing costs.

## Abbreviations

AI: artificial intelligence
AUC: area under the curve
ECAP: Estació Clínica d’Atenció Primària (Primary Care Clinical Station)
ICS: Institut Català de la Salut

## Data Availability

Our manuscript is based on confidential and sensitive health data. However, to support scientific transparency, we will publish deidentified data for reviewers or for replication purposes. The data will be deposited and made available in our publicly accessible Mendeley repository.
