# Correction: Developing an Artificial Intelligence Model for Reading Chest X-rays: Protocol for a Prospective Validation Study

**DOI:** 10.2196/109480

**Published:** 2026-08-26

**Authors:** JMIR Publications Editorial Office

**Affiliations:** 1 JMIR Publications Toronto, ON

Following the publication of “Developing an Artificial Intelligence Model for Reading Chest X-rays: Protocol for a Prospective Validation Study” [1], concerns were raised regarding potential conflicts of interest between reviewer Francesc Lopez Segui and the authors.

These concerns related to prior co-publication with members of the authorship. This information was not disclosed to the JMIR Publications Editorial Office as per journal policy at the time of submission.

This article has been subsequently reassessed by the Editorial Office who determined that the content of the review provided did not unduly influence the decision to accept the article.

As such, the JMIR Publications Editorial Office issues this corrigendum to update the Conflicts of Interest statement:

Reviewer Francesc Lopez-Segui was found to have undisclosed conflicts of interest with members of the author list under the terms of JMIR Publications policy. The handling editor determined that the content of the review did not unduly influence the decision to accept the article.

The correction will appear in the online version of the paper on the JMIR Publications website together with the publication of this correction notice. Because this was made after submission to PubMed, PubMed Central, and other full-text repositories, the corrected article has also been resubmitted to those repositories.
